# A Lossless Multichannel Bio-Signal Compression Based on Low-Complexity Joint Coding Scheme for Portable Medical Devices

**DOI:** 10.3390/s140917516

**Published:** 2014-09-18

**Authors:** Dong-Sun Kim, Jin-San Kwon

**Affiliations:** Embedded Software Convergence Center, Korea Electronic Technology Institute, #68 Yatop-dong, Bundang-gu, Seongnam-Si, Gyeonggi-do 463-816, Korea; E-Mail: jinsan.kwon@keti.re.kr

**Keywords:** biosensor, multichannel bio-signals, joint coding, medical device

## Abstract

Research on real-time health systems have received great attention during recent years and the needs of high-quality personal multichannel medical signal compression for personal medical product applications are increasing. The international MPEG-4 audio lossless coding (ALS) standard supports a joint channel-coding scheme for improving compression performance of multichannel signals and it is very efficient compression method for multi-channel biosignals. However, the computational complexity of such a multichannel coding scheme is significantly greater than that of other lossless audio encoders. In this paper, we present a multichannel hardware encoder based on a low-complexity joint-coding technique and shared multiplier scheme for portable devices. A joint-coding decision method and a reference channel selection scheme are modified for a low-complexity joint coder. The proposed joint coding decision method determines the optimized joint-coding operation based on the relationship between the cross correlation of residual signals and the compression ratio. The reference channel selection is designed to select a channel for the entropy coding of the joint coding. The hardware encoder operates at a 40 MHz clock frequency and supports two-channel parallel encoding for the multichannel monitoring system. Experimental results show that the compression ratio increases by 0.06%, whereas the computational complexity decreases by 20.72% compared to the MPEG-4 ALS reference software encoder. In addition, the compression ratio increases by about 11.92%, compared to the single channel based bio-signal lossless data compressor.

## 1. Introduction

**Figure 1.**
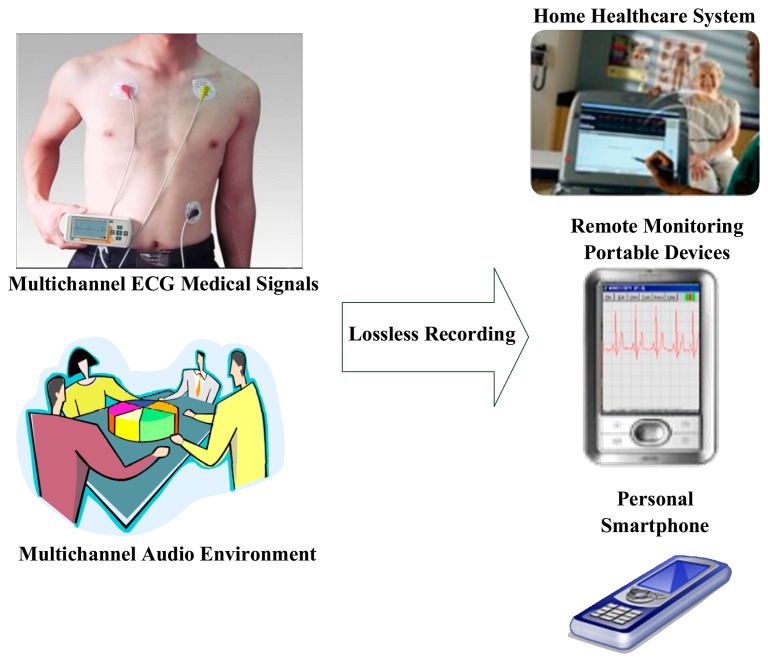
Consumer products for multichannel systems.

The rising demand for recording high-quality audio for mobile products, portable and wireless brain-heart monitoring systems requires a mixed bio-signal lossless data compressor capable of handling multichannel electroencephalogram (EEG), electrocardiogram (ECG) and diffuse optical tomography (DOT) bio-signal data for reduced storage and communication bandwidth requirements [1–3]. For the efficient compression of dynamic biosignals, early predictive coding methods, such as differential pulse code modulation (DPCM), directly encode the amplitude variation of the adjacent sample values. These methods are simple and easy to implement, but the compression rate is relatively low. Run-length coding (RLC) uses the correlation among the symbols for reducing the recording length of each symbol and Huffman codes based on the frequency with which each signal appears are also applied to medical compression systems. These algorithms assign the most economical code length so as to achieve compression. With a flat distribution of the signal in the time domain, the energy will be concentrated on the low-frequency component so the high frequency component can be omitted [4,5]. Most biomedical data compression systems recently use a discrete cosine transform (DCT) with Huffman coder and auto-regression filter with arithmetic coder encode individual channels separately [1]. However, such algorithms have limitations of compression efficiency as the number of simultaneous compression of the monitoring biomedical signals is increased. MPEG-4 ALS, which is a standard lossless compression tool for audio signals based on time-domain prediction, provides a joint channel coding tool for exploiting inter-channel correlations of multichannel signals to improve the compression ratio of multichannel audio streams [6–11]. However, the computational complexity of such a multichannel coding scheme is significantly greater than that of other lossless audio compression algorithms because of additional signal processing such as the weighted subtraction of the residual signals [10]. Consequently, the MPEG-4 ALS encoder requires an embedded processor with more computational power [11] and the heavy computational complexity imposes many constraints on applying the encoder to multichannel audio systems [11–13]. Figure 1 shows a multichannel system that generates suitable compressed streams for various consumer devices. Consumers can record multichannel audio or ECG medical signals with their portable devices in real-time.

In this paper, we propose a low-complexity joint-coding method for multichannel biosignals. The proposed method consists of a joint-coding decision and reference channel selection for avoiding unnecessary joint channel coding processes. We also present a shared hardware architecture for real-time multichannel compression. The rest of the paper is organized as follows: the proposed joint-coding decision method is presented in Section 2 and a hardware implementation of the MPEG-4 ALS encoder is described in Section 3. The performance and computational complexity of the proposed method are evaluated in Section 4. Finally, the implementation results and conclusion are presented in Section 5.

## 2. Low-Complexity Joint-Coding Method for MPEG-4 ALS Hardware Encoder

The joint-coding method of the MPEG-4 ALS encoder can operate with stereo and multichannel audio signals. It uses the difference between two channels as follows:

(1) $$d(n)=x_{2}(n)-x_{1}(n),\;n=0,1,\ldots N-1$$

where *x_1_*(*n*) and *x_2_*(*n*) are the joint stereo or multichannel and *N* is the number of samples in a frame.

Because the difference provides the highest compression ratio when the input signals of the joint channels are equal, the joint-coding performances are then compared to the cross correlation of the joint channels for each frame [11]. To evaluate the performance of the joint-coding method, the compression, *C*, can be defined as:

(2) $$C=\frac{\text{compressed file size}}{\text{uncompressed file size}}\times 100\%$$

**Figure 2.**
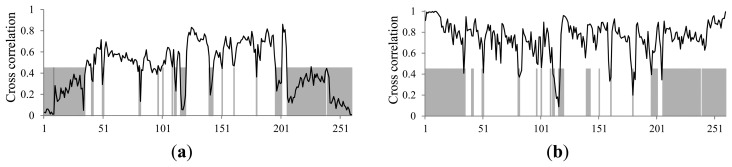
Relationship between compression ratio of joint-coding method and cross correlation: (**a**) residual signal and (**b**) input signal of joint channel.

**Figure 3.**
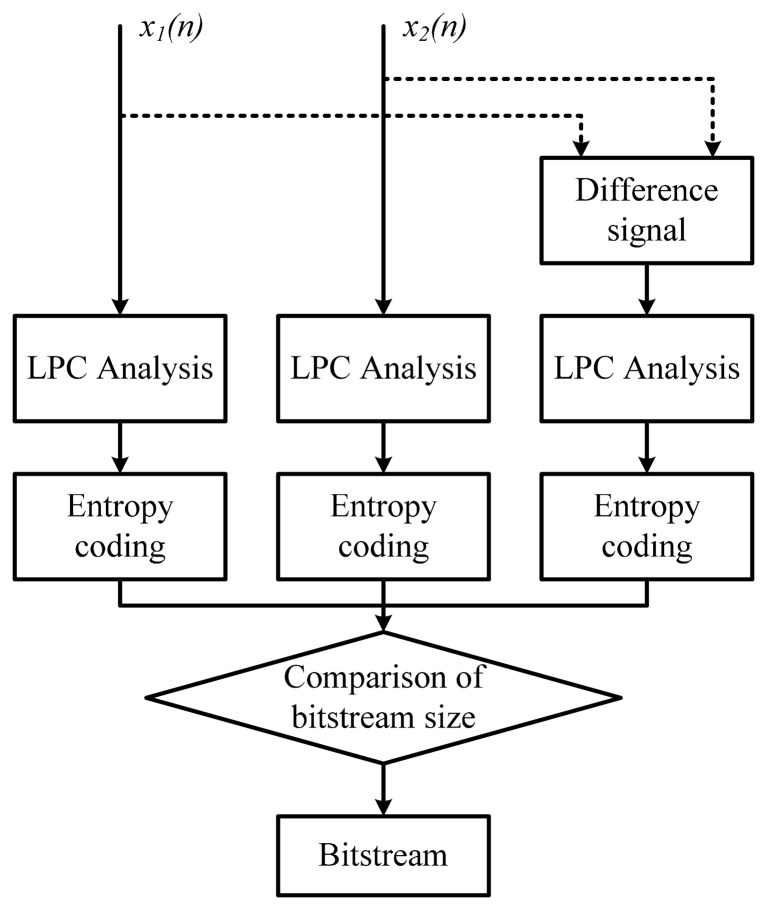
Joint-coding structure of MPEG-4 AL2S reference encoder.

In Equation (2), a smaller *C* implies better encoder performance in terms of the compression ratio. Figure 2a,b shows the performance of the joint-coding method and cross-correlation values for the residual signal and input signals of the joint channel, respectively. The residual signal of the joint channel is obtained by the short-term prediction filter and the formant of the input signal can be removed by the filter. In Figure 2, the gray region indicates the performance of the joint coding method and the black line shows the cross-correlation value of the joint channels. In the gray region, the compression ratio can be improved by joint coding, whereas it is not occur in the other areas. Figure 2a also shows that the compression ratio of the encoder is improved by using the joint-coding method when the residual signals of the joint channel have a high cross-correlation value. However, there is no relation between the joint-coding method and the cross-correlation value of the joint channel input signal to the encoder performance as shown in Figure 2b. Therefore, it can be concluded that the joint-coding performance can only be estimated by the cross-correlation value of the residual signal. We can also define the cross correlation of the residual signals as the decision factor for joint coding. In the MPEG-4 ALS reference encoder, the two input signals and the difference between them are compressed for joint coding as shown Figure 3. The bit stream sizes of the three signals are then compared in the joint-coding method of the MPEG-4 ALS reference encoder, and the signal with the largest bit stream size is removed to ensure a high compression ratio. However, this additional compression process of the different signals increases the overall computational complexity of the encoder by about 50% compared to independent channel encoding methods.

### 2.1. Joint-Coding Decision Method

**Figure 4.**
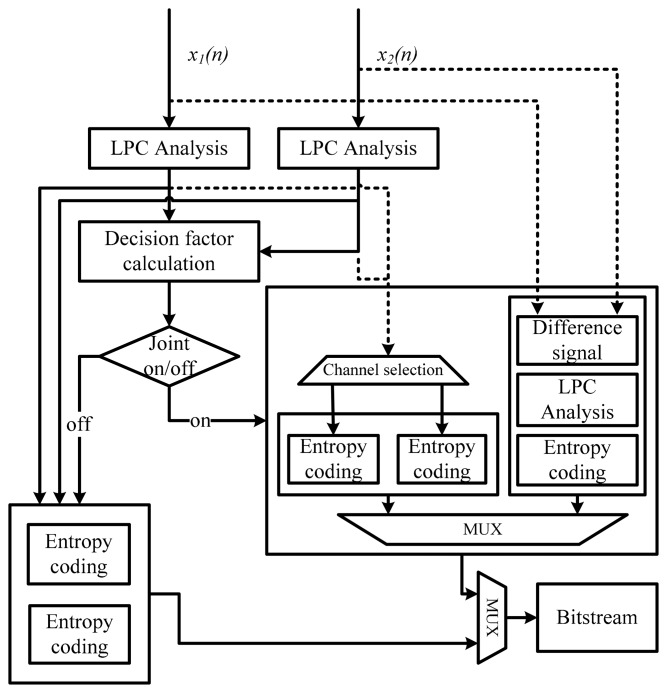
Proposed low-complexity joint-coding structure.

To reduce computational complexity, we propose a joint-coding decision method based on the cross-correlation value of residual signals. As discussed above, the compression ratio of the joint-coding scheme tends to improve when a high cross-correlation value is obtained between the residual signals of the first and second audio channels. Therefore, the cross correlation of the residual signals can be used to estimate the improvement of the compression ratio without an additional compressing process of difference signals and comparing the bit stream sizes as shown in Figure 4. The proposed method requires only an additional calculation of the cross correlation because the generation of linear prediction coefficients (LPCs) and a short-term prediction filter should be processed for the MPEG-4 ALS encoder before entropy coding. The cross-correlation decision factor, *DF*, of two channels' residuals can be calculated by:

(3) $$DF=\frac{\left|\sum_{n=0}^{N-1}e_{1}(n)\times e_{2}(n)\right|}{\sqrt{\sum_{n=0}^{N-1}e_{1}^{2}(n)}\times \sqrt{\sum_{n=0}^{N-1}e_{2}^{2}(n)}}$$

where *e*_1_(*n*) and *e*_2_(*n*) are the residual signals of the joint channel, *N* is the number of samples, and *n* is the sample index in the frame. The joint-coding decision method can be used only when the cross correlation is higher than a predefined threshold and two channels are independently processed when the cross correlation is lower than the following threshold:

(4) $$\begin{array}{l} \begin{array}{cc} if & DF\geq TJ; \end{array}\\ \begin{array}{cc} & \text{joint}-\text{coing} \end{array}\\ \begin{array}{cc} else; & \end{array}\\ \begin{array}{cc} & \text{independent}-\text{processing} \end{array} \end{array}$$

where *TJ* indicates the predefined threshold for the joint-coding decision. Because *DF* is the cross-correlation value of residuals between two signals, the compression ratio of the joint-coding is improved when *TJ* is less than at least 0.5 as shown in Figure 2. But very small *TJ* values significantly increase the computational complexity of the encoding process without improvement of compression ratio. For this reason, the optimum *TJ* value of 0.45 is decided by comparing compression ratios and processing time of MPEG-4 ALS conformance test files [12].

### 2.2. Reference Channel Selection

For joint coding, MPEG-4 ALS encoders compress one reference channel signal and the difference between two input signals. According to the MPEG-4 ALS standard, the reference channel is selected by comparing the bit stream sizes of the two channels after entropy coding. Therefore, the selected channel has a smaller bit stream size than the other channels. To reduce the hardware complexity through the selection operation of the reference channel for joint coding, we use the Rice code and Block Gilbert Moore code (BGMC) [4] for choosing the reference channel before entropy coding. For MPEG-4 ALS entropy coding, the bit stream size of the residual signal can be estimated from the Rice parameter, which determines the required data size to encode one sample [8,9]. The average residual signal, *AbsMean*, for the Rice parameter is calculated as follows:

(5) $$\text{AbsMean}=\frac{\sum_{n=0}^{N-1}|e(n)|}{N}$$

where *e*(*n*) is the residual signal and *n* is the index of the sample in the frame. The proposed method selects the reference channel for joint coding based on the average of each frame. If the reference channel has a lower average value than other channels, a low Rice parameter can occur owing to the low bit stream size. Therefore, the reference channel is selected without entropy coding for two channels as shown in Figure 4.

## 3. Hardware Implementation of a Low-Complexity MPEG-4 ALS Encoder

**Figure 5.**
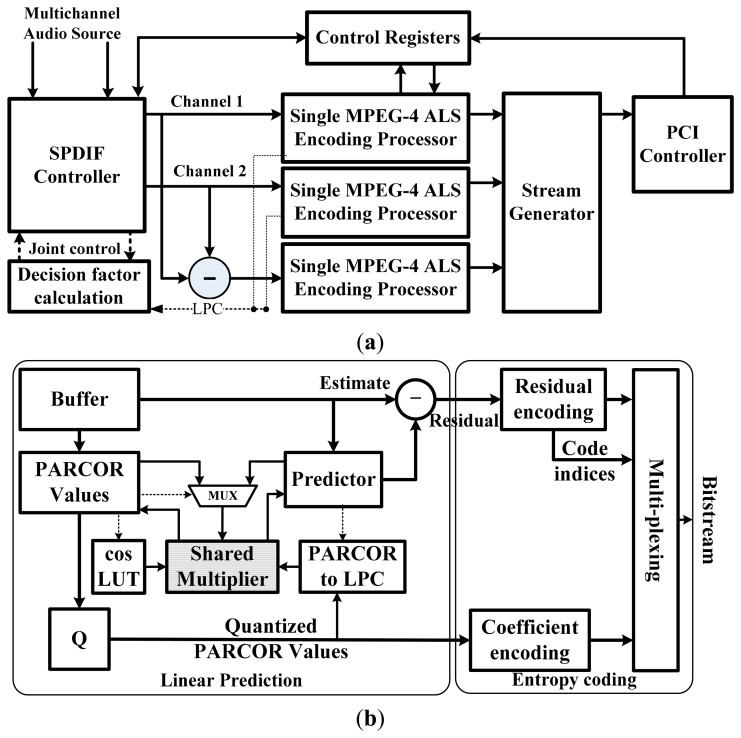
Block diagram for MPEG-4 ALS encoder: (**a**) architecture of real-time multichannel MPEG-4 ALS hardware encoder and (**b**) single MPEG-4 ALS encoding processor.

Figure 5a shows a block diagram of the MPEG-4 ALS hardware encoder for a multichannel audio system. It consists of three MPEG-4 ALS encoding processors for parallel multichannel data compression, an SPDIF audio input digital controller, and a PCI controller for storing encoded streams. For low-complexity MPEG-4 ALS encoder hardware, the reference channel is selected for the joint-coding block with hardware to calculate a decision factor before entropy coding. The proposed method selects the reference channel that has a lower average channel value than other channels. In addition, shared architecture is used for arithmetic operations of partial autocorrelation (PARCOR) value calculation and prediction block. A single MPEG-4 ALS encoder mainly consists of linear prediction and entropy coding blocks as shown in Figure 5b. A buffer stores one frame of original audio samples and a suitable set of PARCOR coefficients are calculated for every frame. The quantized PARCOR values are entropy coded and converted to linear prediction coefficients (LPCs) for short-term prediction. The residual values and Rice parameters are also entropy coded by the Rice coding algorithm.

### 3.1. Linear Prediction

**Figure 6.**
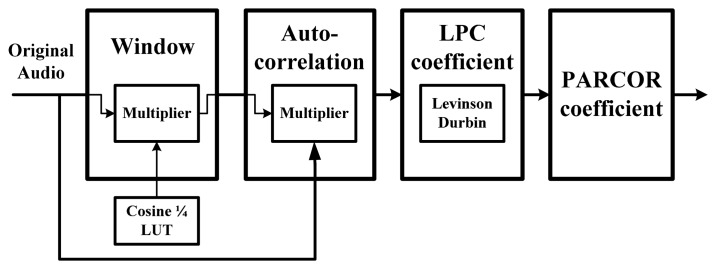
Flow of predictor coefficient calculation.

**Figure 7.**
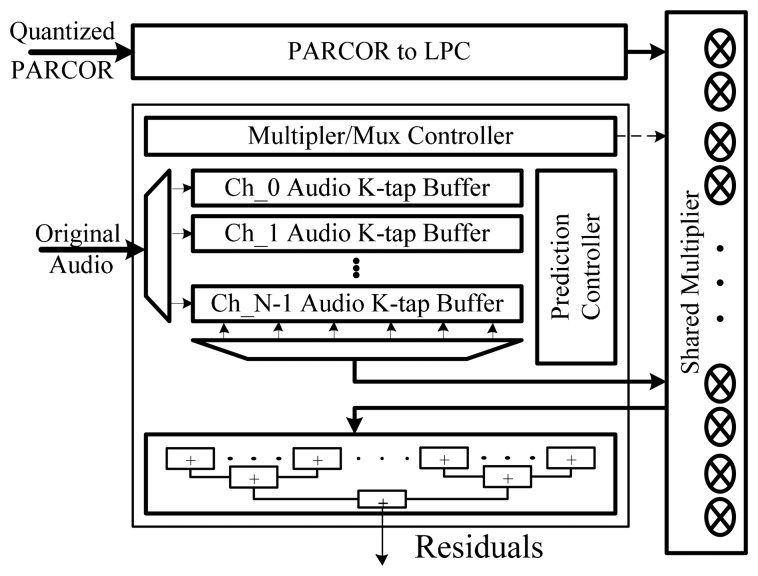
Block diagram of residual calculations.

The MPEG-4 ALS encoding algorithm is based on a forward-adaptive linear prediction method. Linear prediction methods provide estimates of the prediction parameters that minimize the errors between the input value and the predicted value from a number of past samples. In forward linear prediction, the optimal predictor coefficients are initially estimated for each block by the autocorrelation method as shown in Figure 6. This method, which is based on the Levinson-Durbin algorithm in the MPEG-4 ALS standard, has the advantage of providing a simple means to iteratively adapt the order of the predictor [8,11]. After calculating LPCs, the values are used for short-term prediction and are generally implemented using a finite impulse response (FIR) filter. The MPEG-4 ALS standard supports the maximum LPCs order up to 1023 with a bit resolution of 32-bit PCM. To calculate the window data, a Hanning window block with a cosine coefficient indexing scheme is used to reduce the size of a cosine look-up table (LUT). This scheme, which is based on symmetrical characteristics, can reduce the memory size by about 25%. Calculation of the window data can be expressed by:

(6) $$\text{WindowData}[n]=X[n]\cdot (0.5-0.5\cdot \cos(\frac{2\pi n}{N-1})$$

where *X*[*n*] is the original audio sample and *n* is the number of samples in a frame. After calculating window data, the auto-correlated signal is obtained by the auto-correlation block with multipliers and adders. As shown in Figures 6 and 7, we devised a shared architecture for low-complexity encoder hardware and proposed architecture with a common configurable multiplier block. Such a shared multiplier block consists of 16 multipliers and each multiplier is implemented with the radix-4 booth algorithm. To compute the PARCOR value after auto-correlation, the Levinson-Durbin algorithm is used as follows:

Initialization, set *E*_0_ = *R_yy_*(0), *i* = 0, *a*_0_ = 1

(1) Increment *i* by one and calculate:
  (7) $$k_{i}=-(\sum_{j=0}^{i-1}a_{j}^{i-1}R_{yy}(i-j))/E_{i-1}$$
(2) Calculate for *j* = 1, 2, …, *i* − 1:
  (8) $$a_{j}^{i}=a_{j}^{i-1}+k_{i}a_{i-j}^{i-1}$$
(3) Calculate:
  (9) $$E_{i}=(1-k_{i}^{2})E_{i-1}$$
(4) If *i < M*, return to process (2).

where *R_yy_* is the auto-correlated signal, *a_i_^k^* corresponds to the *i-*th coefficient of the *k-*th filter order, and *E* is the calculated prediction coefficient. Direct quantization of the predictor coefficient is inefficient because small quantization errors may result in large deviations [8]. For this reason, the coefficients are quantized based on the following function:

(10) $$\begin{array}{l} {\text{index}}_{1}=\lfloor 64\times (-1+\sqrt{2}\sqrt{{\text{parcof}}_{1}+1}\rfloor \\ {\text{index}}_{2}=\lfloor 64\times (-1+\sqrt{2}\sqrt{-{\text{parcof}}_{2}+1}\rfloor \\ {\text{index}}_{k}=\lfloor 64\times {\text{parcof}}_{k}\rfloor \\ -64\leq {\text{index}}_{n}\leq 63 \end{array}$$

The PARCOR to LPC block has an arithmetic integer function for conversion between quantized values and direct predictor coefficients. A prediction filter is used to remove the envelope of the audio sound, which is a predictable signal, and thus obtain the residual of the audio sound. The predictor calculates the residual value by using an FIR filter. The residuals are calculated with the original audio data and LPCs in the residual calculation block, which consists of the coefficient buffer, prediction controller and shared multiplier as shown in Figure 7.

### 3.2. Entropy Coding

The residual values have smaller amplitude than the original audio values and the amplitude of the residual values are entropy coded using Rice code for low-complexity encoding. The PARCOR coefficients are also compressed with Rice code and indices of the applied codes must be calculated for compressing these coefficients. The entropy coding block mainly consists of two blocks such as the encoder for quantizing PARCOR coefficients and the residuals as shown in Figure 5b. The Rice encoder uses the Rice parameter calculated by the mean of the residual values, and the Rice code is defined by parameter *s* ≥ 0. For a given value of *s*, each code word consists of a *p*-bit prefix and a *s*-bit sub-code. The prefix is denoted by *p* − 1 “1”-bits and one “0”-bit, with *p* depending on the coded value. For a signal value *x* and *s* > 0, *p* − 1 and sub-code are calculated as follows:

(11) $$\begin{array}{l} p-1=\{\begin{array}{ll} x/2^{s-1} & \text{for}\;x\geq 0\\ (-x-1)/2^{2-1} & \text{for}\;x<0 \end{array}\\ \text{sub}=\{\begin{array}{ll} x-2^{s-1}(p-1)+2^{s-1} & \text{for}\;x\geq 0\\ \left(-x-1)-2^{s-1}(p-1\right) & \text{for}\;x<0 \end{array} \end{array}$$

For *s* = 0, there is no sub-code and *p* − 1 is calculated as follows:

(12) $$p-1=\{\begin{array}{ll} 2x & \text{for}\;x\geq 0\\ -2x-1 & \text{for}\;x<0 \end{array}$$

If the *sb_part* flag in header is set, all residual values can be encoded with the same Rice parameter. The residual block is divided into four sub-blocks, which in turn are encoded with a different Rice parameter [6].

**Figure 8.**
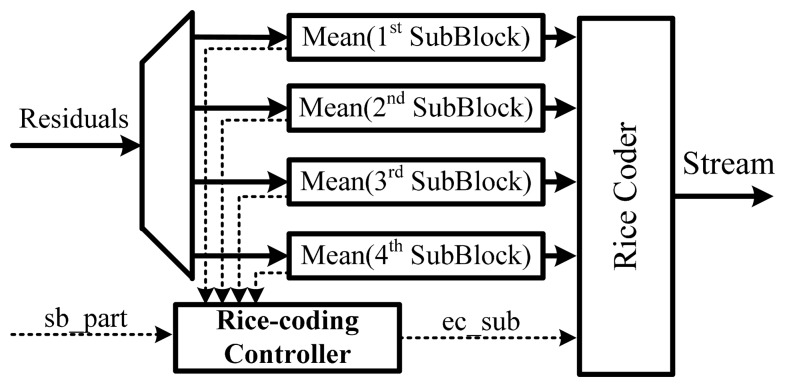
Residual entropy coding.

Given the many different ways to determine the Rice parameter for a given frame data, the MPEG-4 ALS encoder must select suitable parameters by using the mean residual values of the sub-block as shown in Figure 8. To generate the encoded stream, a multiplexing unit combines the encoded prediction coefficient, code indices, encoded residuals and additional information.

## 4. Experimental Results

To evaluate the proposed low-complexity joint-coding method, we use two MPEG-4 ALS conformance test files [14] and three pop music files. Two conformance files are recorded at 48 kHz 16 bit stereo and the other is generated with a 96 kHz 24-bit 6-channel audio source. The proposed low-complexity joint-coding method is compared to the least mean square-recursive least square (LMS-RLS), multi-channel coding, and joint coding method with respect to the compression ratio and encoding time. The compression ratio is evaluated as in Equation (2) and the evaluation results of the compression ratio and encoding time are shown in Tables 1 and 2, respectively. Note that the decision threshold *TJ* is predefined based on the analysis in Section 2. As shown in Table 1, the LMS-RLS method exhibits the highest performance in terms of the compression ratio. However, this method requires too much time for practical use in audio applications. The proposed joint-coding method shows the smallest encoding time and it reduces the encoding time by 20.72% compared to the conventional joint-coding method. The compression ratio of the proposed method is increased by 0.06% compared to the MPEG-4 ALS reference encoder. In addition, the real-time multichannel MPEG-4 ALS encoder described in the previous sections is designed with shared hardware technique and implemented for a field-programmable gate array (FPGA) platform. The implemented multichannel MPEG-4 ALS encoder is operated at 40 MHz clock frequency and includes a 2 KB ROM for cosine LUT and a 17 KB SRAM for the data buffer. Table 3 shows the implementation results of the MPEG-4 ALS encoder. It requires 83 ms for encoding 6-channel audio stream per one second. Hardware efficiency can be defined as the gate count ratio between encoders by using shared and separated multiplier schemes. From these results, we conclude that the proposed architecture reduces the hardware complexity by 13%. To evaluate the compression efficiency on bio-medical signals, we compare a compression ratio with other bio-signal compression algorithm [15,16] as shown in Table 3. The results show that the compression ratio of ECG and EEG data are increased by 15.52% and 8.32%, respectively.

**Figure 9.**
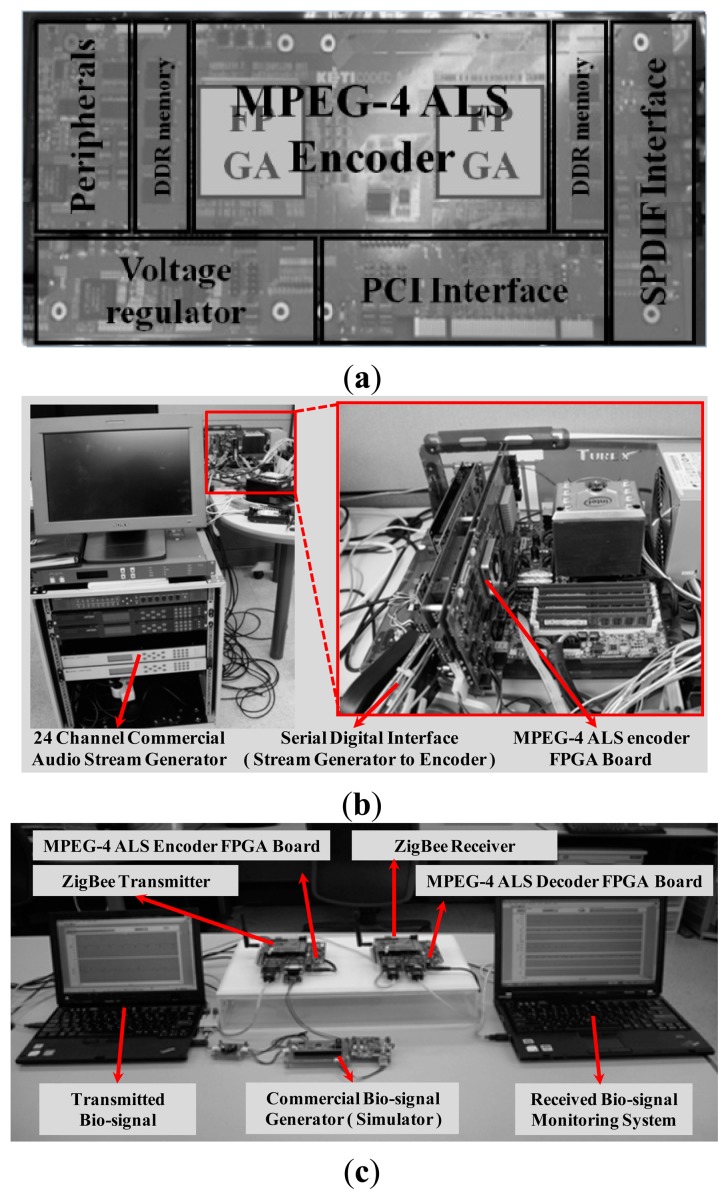
Implemented prototype system: (**a**) real-time multichannel MPEG-4 ALS encoder FPGA board; (**b**) prototype for multichannel audio system and (**c**) a prototype of bio-signal monitoring system.

A prototype real-time multichannel MPEG-4 ALS encoder, which consists of an SPDIF multichannel signal input interface, FPGA for implementing a MPEG-4 ALS encoder hardware, DDR memory, and PCI interface, is shown in Figure 9a. A MPEG-4 ALS prototype encoder processor and system peripheral controllers such as SPDIF and PCI have been implemented with FPGA. The main functions of the MPEG-4 ALS encoder system are verified by using a multichannel audio test platform as shown in Figure 9b. Figure 9c shows a bio-signal monitoring system and it transmits bio-signals through MPEG-4 ALS encoder using ZigBee communications. Four channel ECG and EEG signals are generated from bio-signal generator and these signals are real-time compressed by FPGA proposed joint-coding compression hardware. Such compressed bio-signals are transmitted at the data rate of 256 kbps and received at the monitoring system. Experimental results show that the proposed low-complexity MPEG-4 ALS encoder system can be applied in various low bandwidth communication and low storage space portable devices because of its advantage in compression ratio.

## 5. Conclusions

In this paper, we have proposed a low-complexity joint-coding method for increasing compression efficiency of multichannel biosignals. The proposed method is based on the structure of entropy coding and the interrelationship between the cross correlation of the residual and compression ratios. We also implement a hardware lossless compression module using FPGA platform board for real-time healthcare monitoring systems. The hardware encoder operates at a 40 MHz clock frequency and supports two-channel parallel encoding for the multichannel system. Experimental results show that the compression ratio increases by 0.06%, whereas the computational complexity decreases by 20.72% compared to the MPEG-4 ALS reference software encoder. In addition, the compression ratio increases by about 11.92% compared to the single channel based biosignal lossless data compressor.

## Figures and Tables

**Table 1.** Compression ratio of the proposed MPEG-4 ALS encoder.

**Test Files**	**Channel**	**MPEG-4 ALS Reference Encoder (%)**			Proposed MPEG-4 ALS Encoder (%)*	

		**LMS-RLS**	**Multi-Channel Coding**	**Joint Coding**	**Joing-Coding Decision**	**Joint-Coding Decision + Reference Channel Selection**
Conformance 1	6	36.71	37.11	37.14	37.14	37.14
Conformance 2	2	27.18	28.76	29.02	29.02	29.04
Music 1	2	54.27	56.60	56.76	56.80	56.80
Music 2	2	58.30	61.45	60.76	60.78	60.79
Music 3	2	53.70	55.87	55.91	56.01	55.99
Average	-	46.03	47.96	47.92	47.95	47.95

* *TJ* = 0.45.

**Table 2.** Encoding time of the proposed MPEG-4 ALS encoder.

**Test Files**	**Channel**	**MPEG-4 ALS Reference Encoder (%)**			Proposed MPEG-4 ALS Encoder (%) *	

		**LMS-RLS**	**Multi-Channel Coding**	**Joint Coding**	**Joing-Coding Decision**	**Joint-Coding Decision + Reference Channel Selection**
Conformance 1	6	73.96	2.58	1.11	0.76	0.76
Conformance 2	2	13.16	0.47	0.23	0.17	0.16
Music 1	2	123.16	4.40	2.15	1.61	1.59
Music 2	2	202.48	7.20	3.44	3.27	3.10
Music 3	2	128.27	4.62	2.19	1.62	1.62
Average	-	108.206	3.854	1.824	1.486	1.446

* *TJ* = 0.45.

**Table 3.** Implementation results of the MPEG-4 ALS hardware encoder.

**Feature**		**Implementation Results**	
Average Encoding Time (6 channel/1 s)		83 ms	
Main Clock Frequency		40 MHz	
Memory		ROM (2 KB)/SRAM (17 KB)	
Gate Count (A) (shared multiplier architecture)		101,671 Logic Elements (LEs)	
Gate Count (B) (MPEG-4 ALS reference architecture)		115,879 LEs	
Hardware efficiency (A/B)		0.877	
Compression Ratio (CR)	ECG	Huffman [15]	2.23
		This Work	3.41
	EEG	Karhunen-Loeve [16]	2.8
		This Work	3.65
